# STARD1 and NPC1 expression as pathological markers associated with astrogliosis in post-mortem brains from patients with Alzheimer's disease and Down syndrome

**DOI:** 10.18632/aging.102641

**Published:** 2020-01-05

**Authors:** Fabian Arenas, Fernanda Castro, Susana Nuñez, Gemma Gay, Carmen Garcia-Ruiz, Jose C. Fernandez-Checa

**Affiliations:** 1 Department of Cell Death and Proliferation, Institute of Biomedical Research of Barcelona (IIBB), CSIC, Barcelona, Spain; 2 Liver Unit, Hospital Clinic I Provincial de Barcelona, Instituto de Investigaciones Biomédicas August Pi i Sunyer (IDIBAPS), Barcelona, Spain; 3 Centro de Investigación Biomédica en Red (CIBEREHD), Barcelona, Spain; 4 Research Center for ALPD, Keck School of Medicine, University of Southern California, Los Angeles, CA 90033, USA

**Keywords:** cholesterol, NPC1, StARD1, mitochondria, lysosomes

## Abstract

Alzheimer´s disease (AD) is a progressive neurodegenerative disorder of complex etiology, while Down syndrome (DS) is considered a genetically determined form of AD. Alterations in cholesterol homeostasis have been linked to AD although the role in this association is not well understood. Increased expression of STARD1 and NPC1, which are involved in intracellular cholesterol trafficking, has been reported in experimental AD models but not in patients with AD. Here we analyzed endolysosomal/mitochondrial cholesterol homeostasis, expression of NPC1 and STARD1 and correlation with pathological markers of AD in cortex and hippocampus from post-mortem brains from patients with AD and DS. NPC1 expression was observed in hippocampus from patients with AD and DS. Moreover, STARD1 expression increased in hippocampus and cortex from patients with AD and DS, respectively, and its immunoreactivity discriminated controls from AD or DS with a better accuracy than Aβ_42_. Hippocampal areas stained with the recombinant GST-PFO probe showed increased mitochondrial cholesterol within astrocytes of brains from patients with AD and DS-brains compared to controls. Lysosomal cholesterol accumulation within hippocampal astrocytes was higher in DS than in AD. These data revealed increased intracellular cholesterol loading in hippocampus from patient with AD and DS and suggest that STARD1 could be a potential pre-clinical marker associated with early stages of AD pathology.

## INTRODUCTION

Alzheimer’s disease (AD) is a progressive neurodegenerative disorder of complex etiology and the most common form of dementia, which comprises early-onset AD (EOAD, ~5% of patients) and late-onset AD (LOAD, 95% of patients). The generation of toxic beta amyloid (Aβ) peptides is considered a central player in AD pathogenesis, although other factors such as tau phosphorylation are also key for the progression of AD [1]. While alterations in cholesterol homeostasis have been linked to AD the role for this association is controversial. For instance, there has been evidence indicating that either increased or decreased total brain cholesterol levels are associated with AD-pathogenesis [2]. Whether intracellular cholesterol pools (e.g. endolysosomes and mitochondria) rather than total cholesterol levels are more relevant for AD pathology remains to be established. Indeed, hippocampus and frontal cortex of patients with AD and AD-Tg mice have been shown to exhibit increased expression of the lysosomal cholesterol transporter NPC1 [3], while increased steroidogenic acute regulatory protein (STARD1) expression, which regulates the mitochondrial cholesterol trafficking [4], has been reported in pyramidal hippocampal neurons of patients with AD [5]. Moreover, mitochondrial cholesterol accumulation in a hypercholesterolemic AD-Tg mouse model has been shown to impair mitochondrial antioxidant defense and stimulate oxidative stress, which results in the acceleration of AD-like neuropathology [6, 7]. However, the intracellular cholesterol homeostasis in human AD has not been explored.

Down syndrome (DS) is considered a genetically determined form of AD and individuals with DS invariably develop AD by their fourth decade [8]. Patients with DS exhibit cognitive markers of preclinical and prodromal AD [9]. The progression of AD pathology assessed by neuroimaging and/or cerebrospinal fluid levels of Aβ and tau (total and phosphorylated) revealed a similar pattern between normal population and individuals with DS, although these events in DS occur 10–20 years earlier than in normal population [10, 11]. Aging, obesity and ApoEε4-associated dyslipidaemia are important risk factors for development of AD dementia in normal and DS population [12]. Interestingly, hypercholesterolemic subjects with DS treated with statins exhibit a lower risk factor for AD development that correlates with slower amyloidogenic burden [13]. Besides the overlap between AD and DS, intracellular brain cholesterol homeostasis in patients with DS has not been reported. We hypothesized that alterations in intracellular cholesterol homeostasis in lysosomes and mitochondria in the brain could contribute to the molecular events involved in the AD *continuum*, emerging as a pathological event that could contribute to disease progression. Investigating the intracellular cholesterol homeostasis is critical to improve and expand the therapeutic armamentarium for AD, especially at preclinical stages, given that current clinical trials for AD have been disappointing. In contrast to the controversial role of changes in total brain cholesterol levels in AD [2, 14], our findings on the expression of putative intracellular cholesterol carriers (e.g. STARD1 and NPC1) in patients with AD and DS correlate with disease markers, such as Aβ_42_, and suggest that alterations in intracellular cholesterol homeostasis may be an early molecular event that favours the progression of the AD pathology. Moreover, the accuracy of STARD1 to discriminate controls from AD in the general population and in subjects with DS suggests that StARD1 could be a potential novel marker associated with early molecular events of AD pathology in both populations.

## RESULTS

### Intracellular cholesterol homeostasis in cortex from patients with AD and DS

**Figure 1:**
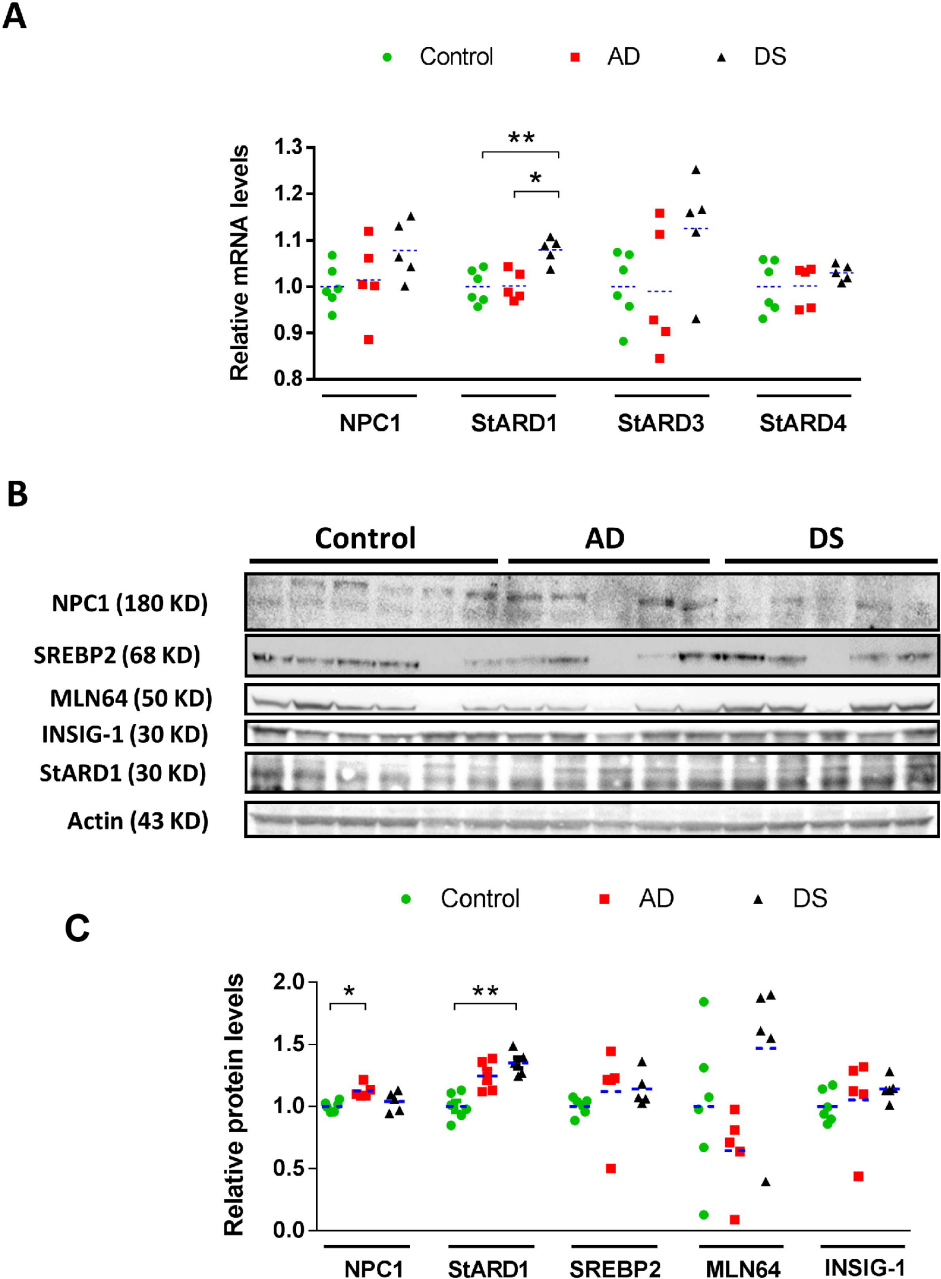
**Cortical expression profile of intracellular cholesterol carriers and sensors/regulators.** (**A**) Relative mRNA levels of NPC1, StARD3, StARD4, and StARD1 in human cortex from AD (n=5), DS (n=5), and control (n=6) subjects. Transcripts levels were normalized with respect to controls using β-actin. (*) p<0.05; (**) p<0.01. (**B**) Immunoblotting of NPC1, StARD1, StARD3/MLN64, mSREBP2 and INSIG-1 of total protein extracts (90 μg/lane) from human cortex from AD (n=5), DS (n=5), and control (n=6) donors. (**C**) Protein levels quantified by densitometry and normalized using β-actin as housekeeping followed by normalization with control group. (*) p<0.05; (**) p<0.01.

As alterations in cortex occur late in AD and reflect disease progression, we first examined the intracellular cholesterol homeostasis in the cortex of sporadic AD and DS using post-mortem cryopreserved cortex samples from patients with AD and DS (Table 1). Transcripts and protein expression of putative cholesterol carriers involved in intracellular cholesterol trafficking (NPC1, STARD3/MLN64, STARD4, and STARD1), as well as sensors/regulators of *de novo* cholesterol synthesis (INSIG-1 and SREBP2) were examined [2] and summarized in Supplementary Table 1. While the expression at mRNA or protein levels of STARD4, INSIG-1 and mature SREBP2 (mSREBP2) seems to be similar between AD, DS and control samples (Figure 1), cortex from patients with DS exhibited a significant overexpression of transcripts for the mitochondrial carrier STARD1 (Figure 1A), which translated at the protein levels (Figure 1B). Moreover, there was a trend for increased mRNA levels of NPC1 and STARD3/MLN64 in cortex from patients with DS (Figure 1A). In addition, cortex from AD-brains exhibited a small but significant increase in the NPC1 protein expression compared to control samples and a trend for increased STARD1 protein levels (Figure 1B). These findings indicate that cortex from patients with DS and AD exhibit increased expression of StARD1 and NPC1, respectively.

**Table 1:** Collected specimens from cases of AD, DS and controls.

	**Subjects (males/females)**						
**Group**	**Cryopreserved Cortex**	**Paraffined hippocampus**	**Cryopreserved hippocampus**	**Total subjects**	**Mean age ± SEM**	**Anatomopathologic diagnosis**	**Clinical diagnosis**
**DS**	5 (3/2)	7 (3/4)	4 (2/2)	7 (3/4)	56 ± 4.31	AD V-VI, CERAD C	Dementia
**AD**	5 (2/3)	7 (4/3)	5 (2/3)	7 (4/3)	80.7 ± 3.00	AD V-VI, CERAD B-C	Dementia
**Control**	6 (4/2)	7 (5/2)	5 (4/1)	7 (5/2)	69.7 ± 4.79	Without neurological lesions (5)	Cognitively healthy
						Frontotemporal degeneration (1)	FTD
						iLBD; incidental Lewy body disease (1)	Cognitively healthy

Note: For immunohistochemical and immunofluorescence analysis, 5 μm thin paraffin sections of hippocampus were available for all human brain samples, while 14 μm thin sections of frozen hippocampus were available for just 19 cases to analyse the intracellular cholesterol levels by immunofluorescence. On the other hand, cryopreserved human cortex of just 16 donors were available to assess gene expression and western blotting analysis. FTD, frontotemporal dementia.

### STARD1 and NPC1 immunoreactivity in hippocampus from patients with AD and DS

**Figure 2:**
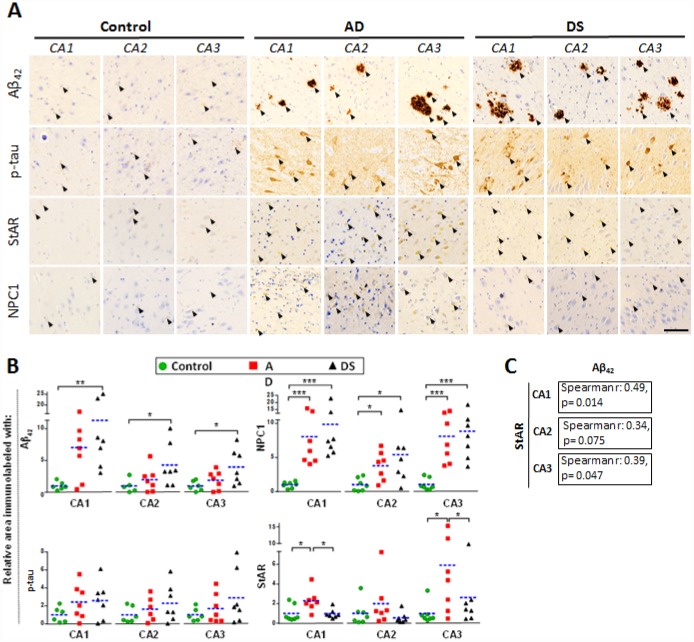
**Hippocampal expression of AD biomarkers and lysosomal/mitochondrial cholesterol carriers.** (**A**) Representative images of immunohistochemistry of paraffin sections (5 μm) against Aβ_42_, p-tau, StARD1 and NPC1 for CA1, CA2 and CA3 hippocampal regions from AD (n=7), DS (n=7), and control (n=7) subjects. Positive immunoreactivity is shown by black arrows. Scale bar: 100 μm. (**B**) Quantitation of IHC shown in A using Image J software as described in Supplementary methods. For each hippocampal region, the % of immunolabeled area was normalized to control group. (*) p<0.05; (**) p<0.01; (***) p<0.001. (**C**) Spearman’s correlation values between IHC-immunolabeling for Aβ_42_ and StARD1 in each hippocampal region.

AD pathology is characterized by atrophy in hippocampus, temporal lobes and parietotemporal cortices where neuronal death correlates with amyloid plaques (largely composed of Aβ_42_) and neurofibrillary tangles (NFTs) [15]. Interestingly, an accelerated Aβ burden with greater plaques deposition and NFT have been described in the hippocampus of DS subjects exhibiting AD pathology [8]. Therefore, we examined the hippocampal expression of cholesterol carriers involved in lysosomal (i.e. NPC1) and mitochondrial (i.e. STARD1) trafficking and compared this outcome with the pathological markers Aβ_42_ and p-tau, summarized in Supplementary Table 1. Immunohistochemical analysis of paraffin-fixed hippocampal sections showed increased Aβ_42_ and p-tau immunoreactivity in patients with AD and DS compared with control subjects (arrows in Figure 2A and 2B). These differences were significant for Aβ_42_ staining in CA1, CA2 and CA3 hippocampal regions from patients with DS, in agreement with the intrinsic overproduction of Aβ in this population [8]. Moreover, there was a trend for increased Aβ_42_ in CA1 region from patients with AD. Interestingly, CA1, CA2 and CA3 hippocampal regions from patients with AD and DS showed a significant higher NPC1 protein expression than control samples (Figure 2A and 2B). In line with previous findings [5], the expression of StARD1 in hippocampal regions revealed a significant increase of STARD1 immunoreactivity in CA1 and CA3 areas from patients with AD compared with DS and controls (Figure 2A and 2B). Moreover, when STARD1 and Aβ_42_ immunoreactivity were compared among all samples, the hippocampal region-specific pattern of STARD1 expression significantly correlated with Aβ_42_ deposition in CA1 and CA3 (Figure 2C). Thus, although AD and DS are two related diseases, each exhibit specific changes regarding the expression of intracellular cholesterol carriers STARD1 and NPC1, with STARD1 overexpression in hippocampus being specific for AD while in cortex the increase seems to be characteristic of DS. In contrast, the increase in expression of NPC1 occurs in both diseases. In addition, the differential turnover between proteins and their corresponding mRNA likely contribute to the specific pattern of expression of STARD1 and NPC1 at the mRNA and protein levels in the cortex between AD and DS samples. Further research will be required to understand the full impact of these changes in AD pathology and its genetically related form DS.

### Discrimination capacity of NPC1 and STARD1 immunoreactivity in hippocampus from patients with AD and DS

**Figure 3:**
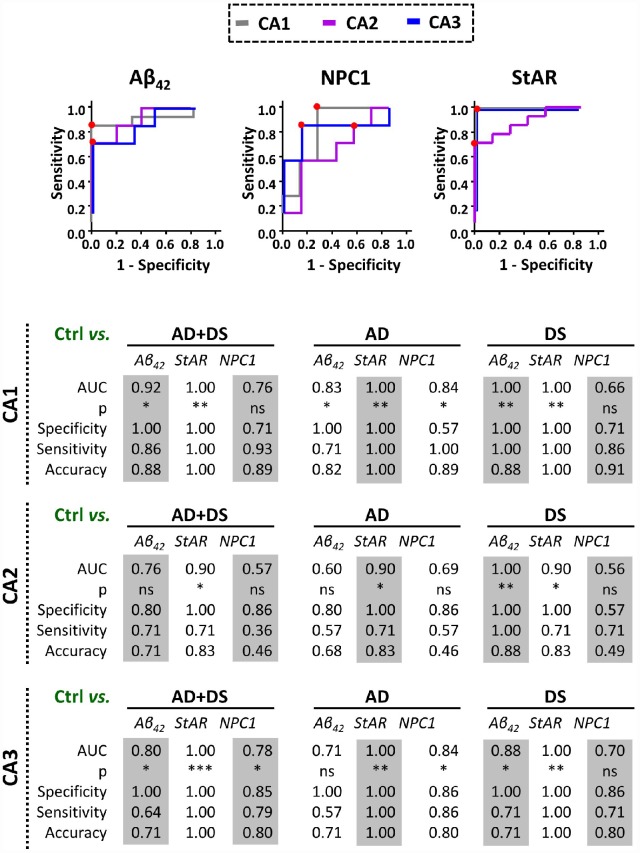
**Discrimination capacity of amyloid accumulation and lysosomal/mitochondrial cholesterol carriers.** ROC curves of immunolabel for Aβ_42_, NPC1, and StARD1 in each hippocampal region with the significantly highest AUC resulted in the comparison between control and AD, DS, or AD+DS groups (Lower panel). Red dots show the cutoff for the corresponding IHC-immunolabel that better discriminate AD and/or DS condition from normal controls in each hippocampal region. (*) p<0.05; (**) p<0.01; (***) p<0.001.

In analogy with Aβ_42_, we searched for expression of both NPC1 and STARD1 in the paraffin-fixed hippocampal samples as potential discrimination factors between AD and/or DS from normal controls. For each hippocampal region we analyzed the relative area immunolabeled with Aβ_42_, NPC1, and STARD1 and performed ROC curves analysis between controls subjects and patients with AD, DS, or the sum of both AD+DS groups (Figure 3). As expected, Aβ_42_ deposition revealed a highly significant AUC with a high accuracy not only to identify AD and/or DS samples as positive (sensitivity), particularly in CA1 hippocampal areas, but also to identify the control subjects (specificity) in all hippocampal regions from DS-brains (Figure 3). Similarly, in hippocampal CA1 and CA3 regions, NPC1 immunolabeling properly discriminated AD individuals, but not DS subjects, from controls (Figure 3). Unexpectedly, STARD1 discriminated AD and/or DS samples from control individuals in all hippocampal regions (Figure 3), suggesting that STARD1 immunolabeling exhibits a greater discrimination capacity than Aβ_42_. Moreover, multivariate data analysis of the cohort by principal components analysis (PCA) showed an efficient visualization of the data for CA1 and CA3, capturing more than 97.6% and 85% of the markers variability, respectively, with a clear separation between control subjects from AD and DS patients (Supplementary Figure 1).

### Amyloid-related hippocampal astrogliosis associated with increased lysosomal and mitochondrial cholesterol content in patients with AD and DS

**Figure 4:**
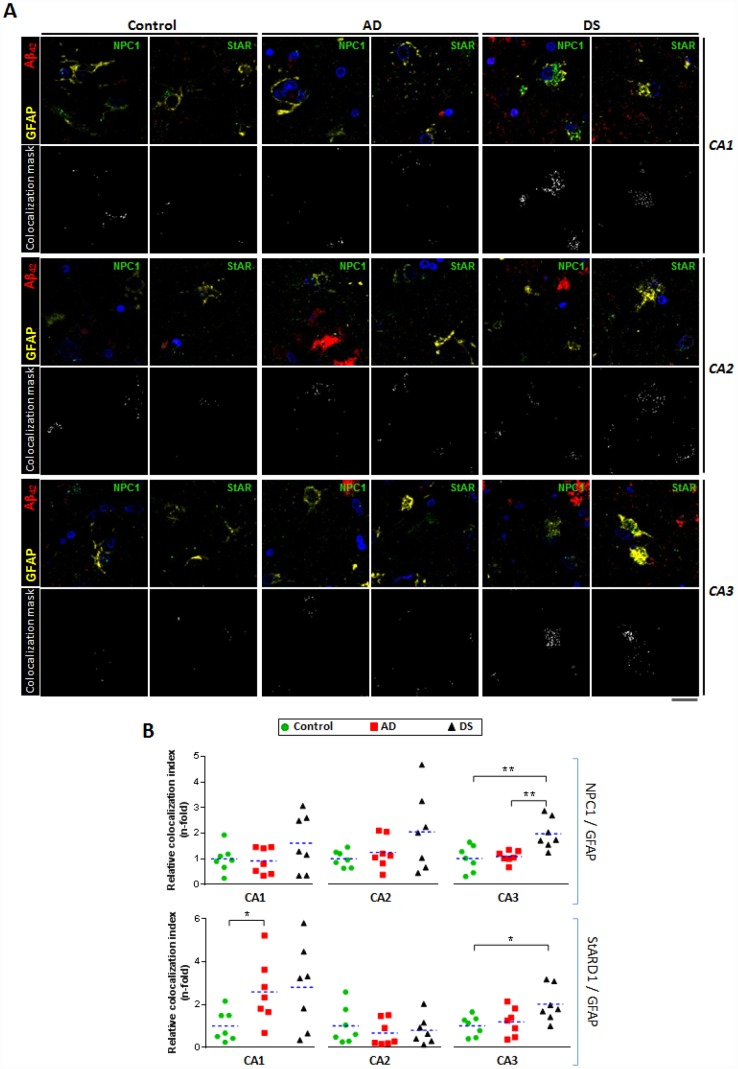
**Astrocytes-expressing lysosomal and mitochondrial cholesterol carriers in hippocampal regions.** (**A**) Representative confocal images of paraffined hippocampal regions (5 μm) from AD (n=7), DS (n=7), and control (n=7) subjects immunolabeled against Aβ_42_ (red), GFAP (yellow), and NPC1 or StARD1 (both green). Nuclei are stained with Hoechst 33342 (blue). Lower panels show the colocalization mask between GFAP and NPC1 or StARD1 (white) highlighted in the squared areas. Scale bar: 10 μm. (**B**) Astrocyte colocalization with NPC1 (lysosomal) and StARD1 (mitochondrial) cholesterol carriers into hippocampal regions from AD, DS, and control subjects. 10 images per hippocampal region and per sample were analysed with Image J to assess the index of astrocyte (GFAP+) colocalization with NPC1 or StARD1. (*) p<0.05; (**) p<0.01; (***) p<0.001.

To investigate whether disruption of intracellular cholesterol homeostasis correlate with AD, we used confocal immunofluorescence imaging to explore the pattern distribution of the intracellular cholesterol transporters NPC1 and STARD1 within the astrocytes surrounding amyloid deposition in the different hippocampal regions (Figure 4). In agreement with previous observations in post-mortem human AD tissues as well as in animal models [16, 17], reactive astrocytes surrounding Aβ_42_ deposition were observed in hippocampus from patients with AD and DS (Figure 4A). Moreover, the colocalization analysis of ten representative images per sample revealed that astrocytes in hippocampus from patients with DS exhibit higher NPC1 expression than in AD and control samples, particularly in CA3 region (Figure 4B), suggesting increased lysosomal cholesterol levels in astrocytes from patients with DS. In addition, the analysis of the STARD1 content in hippocampal astrocytes indicated a region-specific significant increase of STARD1/GFAP colocalization index in CA1 and CA3 areas for patients with AD and DS, respectively, compared to controls (Figure 4B). Moreover, pilot observations following the staining of paraffined hippocampal sections of brains from AD, DS and control groups with NeuN and IBA1 antibodies as neuronal and glial markers, respectively, indicated that STARD1 and NPC1 expression increased in hippocampal neurons from AD and DA brains with respect to control, without changes in glia (not shown).

**Figure 5:**
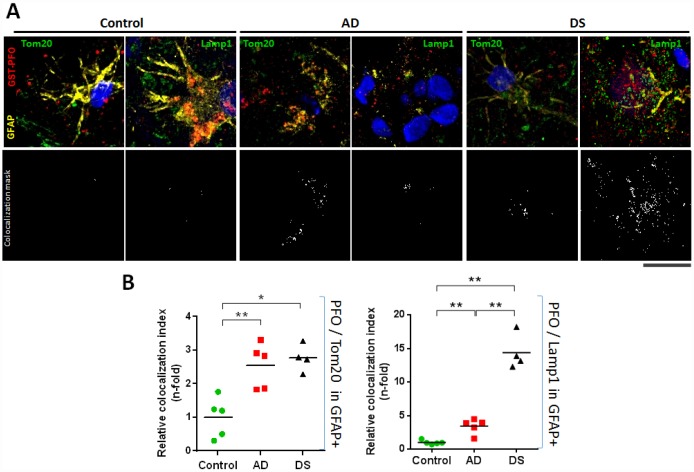
**Lysosomal and mitochondrial cholesterol homeostasis in hippocampal astrocytes.** (**A**) Representative confocal representative images of 4 μm thin-stacked cryopreserved hippocampus from AD (n=5), DS (n=4), and control (n=5) subjects immunolabeled with GST-PFO (red), GFAP (yellow), and Tom20 or Lamp1 (both green). Nuclei are stained with Dapi (blue). Lower panels show colocalization mask (white) between GST-PFO and Tom20 or Lamp1, respectively, highlighted in the squared areas. Scale bar: 10 μm. (**B**) Cholesterol (PFO+) colocalization with mitochondria (Tom20+) or lysosome (Lamp1+) into hippocampal astrocytes (GFAP+) from AD, DS, and control donors. 10 images per sample were analysed with Image J to assess the index of astrocytic cholesterol colocalization with Tom20 or Lamp1. Values are relativized with control to show differences as n-fold. (*) p<0.05; (**) p<0.01.

To assess the intracellular cholesterol distribution within hippocampal astrocytes (i.e. GFAP+), cryopreserved hippocampal samples of patients with AD and DS were analysed for the colocalization between GST-PFO probe, which detects cholesterol in membranes, with Lamp1 or Tom20 immunoreactivity to stain lysosome or mitochondria, respectively (Figure 5; and Supplementary Table 2). In line with the STARD1/GFAP colocalization, we found that the mitochondrial cholesterol content into hippocampal astroglia from patients with AD and DS was >2.5-fold higher than control donors (Figure 5B). Moreover, astroglial lysosomal cholesterol accumulation (PFO/Lamp1 in GFAP+) significantly increased in hippocampus from AD and DS patients compared to controls (Figure 5B), an outcome that was more pronounced in DS (14.41±1.322 fold) than in AD brains (3.43±0.502 fold). Interestingly, these findings correlated with the increased Aβ_42_ accumulation in the CA1, CA2 and CA3 hippocampal regions of DS-brains (Figure 2B). Furthermore, PCA analysis revealed that the levels of astroglial NPC1 and STARD1 content as well as the intraorganelle-cholesterol levels are able to separate each group (Supplementary Figure 2).

## DISCUSSION

We have analyzed the intracellular cholesterol trafficking in post-mortem samples from patients with DS and AD and assessed whether this event correlated with AD markers. An intriguing finding was that Aβ_42_ but not p-tau immunoreactivity significantly increased in CA1-3 hippocampal regions only in patients with DS, with a trend for increased Aβ_42_ levels in CA1 from patients with AD. These findings in DS are consistent with the intrinsic Aβ overproduction in these subjects [8]. Moreover, neuropathological analysis of amyloid pathology in DS described the onset of mid-severity changes in hippocampal CA1 region at 35 years of age, in which Aβ_42_ deposition occurred in the absence of tau pathology [18]. Conversely, tauopathies and the presence of NFTs that commonly coexist with LOAD can occur without amyloid pathology [19, 20]. In addition, it has been suggested that the earliest appearance of tau pathology in AD occurs in the *locus caeruleus* instead of hippocampus [21], which could account for the lack of p-tau immnunoreactivity in hippocampal samples from our cohort of patients with AD. While the present work is the first to our knowledge to address the expression of putative intracellular cholesterol carriers (e.g. STARD1 and NPC1) in AD and DS and their correlation with pathological markers, the role of changes in total cholesterol levels in AD is controversial [2, 14], consistent with the unsettled impact of statins in the prevention or treatment of the disease, which will require further randomized controlled trials [22, 23].

Although the groups studied were not age-matched given the limited number of samples and the life-expectancy of individuals with DS [8], we performed the comparisons with AD and controls considering AD pathogenesis as a function of age in DS, a population that exhibit a high risk to develop AD-type dementia [12, 24]. Moreover, as described previously [18], individuals with DS developed signs of AD-pathology by 50-55 years of age, which coincides with the age range of the subjects with DS analyzed in our study (Table 1). In this context, interestingly we found increased expression of NPC1 and STARD1 in cortical samples from patients with AD and DS, respectively, (Figure 1). A link between lysosomal cholesterol and Aβ_42_ deposition in neurodegeneration has been described in Niemann–Pick type C (NPC) disease, a rare autosomal recessive disease associated mostly with NPC1 mutations, characterized by lysosomal accumulation of unesterified cholesterol [25]. While the etiology and epidemiology of AD and NPC differ, these neurodegenerative diseases share many disease-related molecular pathways, including cholesterol accumulation, lysosomal abnormalities, tau hyperphosphorylation, APP processing and Aβ deposition [2, 25]. Moreover, while increased mRNA and protein levels of NPC1 have been described within the hippocampus of both patients with AD and mice [3], the hippocampal expression pattern of NPC1 in human DS-brains has not been reported. Our findings suggest that while altered hippocampal NPC1 expression could be a common feature of DS and AD (Figure 2), the NPC1 immunolabeling as a disease marker of AD does not discriminate AD-pathology in patients with DS from the controls (Figure 3), suggesting that the AD *continuum* in the hippocampus of subjects with DS differs from NPC disease.

Cholesterol is known to promote the amyloidogenic processing of APP, which occurs predominantly in lipid-rafts where most components of the amyloidogenic machinery, such as APP, Aβ, BACE1, presenilins (PSEN1/2), and γ-secretase are present [26, 27]. Moreover, cholesterol is thought to interfere with the α-secretase-dependent non-amyloidogenic APP processing [28], and PSEN1/2 and γ-secretase have been localized at the mitochondrial ER-associated membrane (MAM) [29, 30]. Interestingly, although mitochondrial membranes are low in cholesterol, the pool of cholesterol of the mitochondrial inner membrane (MIM) is crucial for the synthesis of neurosteroids to maintain physiological GABAergic responses that modulate memory function, which is particularly affected in subjects with DS [31, 32]. The STARD1-dependent trafficking of cholesterol to MIM is the rate-limiting step for steroidogenesis and changes in mitochondrial cholesterol levels can impact this process and affect mitochondrial antioxidant defense [2, 5–7, 33]. However, the role of STARD1 in human AD is poorly understood and has not been examined in DS. We show for the first time that the hippocampal overexpression of STARD1 correlates with amyloid deposition suggesting a relationship between the accumulation of mitochondrial cholesterol and the development of AD-pathology (Figure 2). Unexpectedly, our findings in hippocampal samples from patients with DS and AD suggest that STARD1 immunolabeling can act as a pathological mark of AD even better than Aβ_42_ deposition in post-morten samples (Figure 3), implying that the hippocampal alterations in the expression of STARD1 could occur in a temporal pattern parallel to the pathogenic amyloidogenic burden. Whether these alterations in STARD1 occur before Aβ_42_ deposition in human AD requires further investigation. However, previous evidence in experimental models pointed that enhanced mitochondrial cholesterol levels in AD mouse models have been shown to sensitize neurons to Aβ-induced inflammation and toxicity by depleting mGSH by an ER stress-dependent mechanism [6, 7, 33]. Moreover, as Aβ-induced ER stress is considered an indirect effector of the Aβ-neurotoxicity in early stages of AD [34], our data suggest that STARD1 overexpression in the hippocampus may be a potential early molecular event associated with AD, as strengthened by the increase seen in DS, an accelerated genetic form of AD. In this context, it is widely recognized that neuroinflammation in AD is an early pathological-associated response, which is exacerbated with ageing and contributes to disease progression [18, 35].

Although not statistical significant, our findings do suggest a trend for lower expression of STARD3/MLN64 in the cortex of AD samples (Figure 1B), whose impact in regulating the mitochondrial pool of cholesterol remains to be understood. In this regard, it is interesting to note that increased expression of MLN64 has been reported in the lysosomal storage disorder Niemann-Pick type C disease, which correlated with increased mitochondrial cholesterol accumulation [36]. However, it has also been reported that targeted mutations of the MLN64 START domain have been shown to cause minor alterations in metabolism and intracellular distribution of cholesterol [37]. Further investigation will be required to decipher the impact of MLN64 in mitochondrial cholesterol regulation and AD pathogenesis.

Reactive astrogliosis has emerged as a relevant pathological response in the early stages of AD [38], which correlates with cognitive decline [16]. Since astrocytes are the most prevalent cell type in the brain and form an intricate system of connections to control a wide range of brain functions, including regulation of blood-brain barrier, the astroglial atrophy could have far-reaching consequences on synaptic transmission that accounts for the spatio-temporal progression of AD-neurodegeneration [17]. We confirm the presence of reactive astrocytes surrounding amyloid plaque in the hippocampus of AD and DS, and observed a hippocampal region-specific increased expression of STARD1 within astocytes (Figure 4) that correlates with the accumulation of mitochondrial cholesterol in both groups (Figure 5). Similarly, we also found astroglial NPC1 overexpression and astroglial accumulation of lysosomal cholesterol in AD and DS (Figures 4 and 5). These results are in line with findings reporting the activation of rat astrocytes induced by cholesterol exposure *in vitro*, resulting in enhanced APP/BACE-1 interaction within lipid-rafts, increased APP content, and enhanced ROS production [39]. Moreover, recent observations from a single cell brain atlas of human AD indicated the diversity of astrocytes in AD under the control of the transcription factor EB, a master regulator of lysosomal function, which initiated a regulatory cascade containing multiple AD GWAS genes [40]. Taken in consideration that subjects with DS display abnormal lipid metabolism from the embryonic stages [41] and that *SOD1*, *BACE-2*, *S100β* and *APP* genes are located at HSA21 [42], it is conceivable that subjects with HSA21 trisomy could have a basal cholesterol-mediated astrocyte activation. These findings along with the observed increased NPC1 expression in DS individuals (Figure 2) suggests that an enhanced hippocampal lysosomal cholesterol trafficking could result in increased mitochondrial cholesterol loading in hippocampal areas, possibly coinciding with signs of early AD-pathogenesis. Moreover, elevated astrogial-STARD1 expression in individuals with AD implies accumulation of mitochondrial cholesterol in activated astrocytes within CA1 independently of NPC1. Whether this outcome reflects that post-mortem samples from patients with AD represent a late stage of the disease masking the early intra-organelle cholesterol mobilization remains to be further investigated.

Finally, whether the increase in hippocampal Aβ_42_ deposits reflects an impaired Aβ clearance by increased lysosomal cholesterol remains to be further confirmed in human neurodegeneration. Although the astrocyte-dependent lipidation of ApoE could contribute to the ability of glia to clear Aβ burden, this event does not seem to occur in ApoE4-carrying subjects [2], and defects in Aβ clearance have been detected in cerebrospinal fluid of >98% of LOAD cases [43], which may reflect defects in the endolysosomal/autophagy system in the early neuropathological stages of AD [44]. In line with this possibility, we have recently reported that elevated intracellular cholesterol levels in a hypercholesterolemic mouse model increased Aβ-induced autophagosome formation, but impaired the fusion with lysosomal vesicles causing deficient autophagy-dependent Aβ degradation [45]. Considering that DS might serve as a model for early AD [12], the observations of increased lysosomal cholesterol loading support the notion that abnormal cholesterol distribution in lysosomes can impact mitochondrial function through sustained mitochondrial cholesterol accumulation and Aβ clearance, emerging as an early event in AD pathogenesis.

In conclusion, this exploratory study in post-mortem brains from patients with AD and DS revealed increased mitochondrial and lysosomal cholesterol levels in hippocampus from patients with AD and DS, which correlate with higher expression of STARD1 and NPC1. Moreover, our findings indicate the ability of STARD1 expression to discriminate controls from AD in the general population and in subjects with DS and suggest that STARD1 could be a potential marker associated with early molecular events of AD pathology. Further studies with a higher number of human samples are needed to validate these findings. In addition, future research using mice with tissue-specific deletion of STARD1 are warranted to define its role in AD pathology.

## MATERIALS AND METHODS

### Human brain samples

Twenty-one cases from the Biobank of Hospital Clinic/IDIBAPS of Barcelona were collected following approval of the Clinical Research Ethics Committee of the Hospital Clinic of Barcelona (HCB/2015/0595). Samples were scored by CERAD scale (A-C) and Braak stage (0-VI) and categorized as AD, DS, and control groups. For each group, demographic data as well as clinical and anatomopathological diagnosis are detailed in Table 1. Please find the BRISQ checklist in Supplementary Methods detailing the biospecimen samples collected from patients with AD and DS.

### mRNA isolation and RT-qPCR

Total RNA was extracted from 150 mg of frozen cortex of human brains, using TRIzol reagent (Invitrogen), cleaned by RNeasy columns (Qiagen), and quantified through Nanodrop spectrophotometer (Thermo-Fisher Scientific). Real-time qPCR was performed using the iScript™ One-Step RT-PCR Kit with SYBR® Green (Bio-Rad) following the manufacturer’s instructions. Briefly, 200 ng of total RNA were mixed with 300 μmol/l of specific primers for each gene assessed (Supplementary Table 3) in 1× reaction buffer at final volume of 10.5 μl. RT-qPCRs were performed in triplicate using a CFX380 Real-Time System (Bio-Rad) following the manufacturer’s instructions. Gene expression values were normalized using actin as housekeeping gene.

### Western blotting

Human frozen cortex (350 mg) were homogenized on ice in anti-proteases/anti-phosphatases (Complete/ PhosSTOP, Roche) containing RIPA lysis buffer. Protein quantitation was assessed by BCA (Pierce) and samples (90 μg/lane) were resolved by 4-12% SDS-PAGE (Bio-Rad), electrotransferred to nitrocellulose membranes (Trans-Blot Turbo, Bio-Rad), followed by 5% BSA-blocking and incubation with primary antibodies (Supplementary Table 4) at 4°C overnight. After washing, membranes were incubated 1 hr at RT with the corresponding horseradish peroxidase-coupled secondary antibodies and visualized using the Pierce ECL Western Blotting Substrate (Thermo Scientific). Immunoblots images were captured using LAS4000 (GE Healthcare) and quantitation of the bands was performed by Image J free software (NIH).

### Immunohistochemistry

Paraffin sections (5 μm) of hippocampus were processed according to the avidin–biotin–peroxidase staining method (Vectastain ABC kit; Vector Lab), as described previously [7] and detailed in Supplementary Methods.

### Recombinant GST-PFO probe

To optimally assess the localization of cholesterol in hippocampal tissues, we generated a recombinant Perfringolysin O (PFO) fusioned with Glutathione-S-Transferase (GST-PFO), as described [46]. In brain tissues, PFO binds and detects cholesterol in membranes when its levels exceed 30% mol, while the fusion with GST allows PFO detection by immunofluorescence. The sequence to design the plasmid was DNA M36704 (NCBI database) corresponding to the gen of PFO in the *Clostridium perfringens* genome. Additionally, the signal peptide was eliminated from the PFO gen to enable the intracellular stance of the protein. The resulted sequence was synthesized into the plasmid pGEX 4T-1 (GenScript) between the *BamHI* and *SmaI* restriction sites*.* The plasmid was engineered to make a fusion protein with the GST tag. After bacterial production, protein extraction and purification, the GST-PFO probe was dialyzed, concentrated, quantified and stored at −80°C.

### Immunofluorescence

Paraffin sections (5 μm) of hippocampus were dewaxed/rehydrated and treated for antigen retrieval as was described above for immunohistochemical analysis. Sections were permeabilized with 0.2% Triton X-100 in blocking buffer (5% goat serum + 1% BSA in PBS) during 10 min, followed by washing ×3 with PBS and incubation overnight at 4°C with primaries antibodies (Supplementary Table 4) into a dark-humid chamber, as described in detail in Supplementary Methods.

### Image analysis

Detection and quantitation of immunoreactivity for Aβ_42_, p-tau, NPC1, and STARD1 was performed from high-resolution scanned immunohistochemical images of hippocampus using the Image J Software after *Color Deconvolution* plug-in [47], as detailed in Supplementary Methods.

### Statistics

Data are shown as mean ± S.E.M and statistical analysis performed with the GraphPad Prism-5 software (San Diego, CA). Due to the number of analysed samples (≤7 for each group), nonparametric tests were employed. Differences between AD, DS and control samples were analysed with Kruskall-Wallis test followed by Mann-Whitney *U*-test to analyze the differences between two groups. The Spearman's rank-correlation coefficient *r_s_* was also determined when pertinent. Two-tailed p values <0.05 were considered statistically significant. ROC curves were generated and the area under ROC curves (AUC) was calculated to evaluate sensitivity and specificity of immunolabeling within hippocampal regions. ROC curves from control group were compared to AD or DS or AD+DS groups. The cut-off points on the ROC curves at which accuracy of disease detection was maximal were selected. Additional visualization of the association between markers and subject status (i.e. control, AD, or DS) was performed through multivariate data analysis by PCA using JMP 9.0 software (SAS Institute Inc.). PCA show the data variability as the covariance of samples for each marker (descriptors) projected into a subspace made of orthogonal linear axis, so-called principal components.

## Supplementary Material

Supplementary Methods

Supplementary Figures

Supplementary Tables
