# Supplementary material for: STARD1 and NPC1 expression as pathological markers associated with astrogliosis in post-mortem brains from patients with Alzheimer's disease and Down syndrome
Source: Aging (Albany NY). 2020 Jan 5;12(1):571–92. doi: 10.18632/aging.102641 (PMC6977657; doi:10.18632/aging.102641)
Supplement: Supplementary Methods [file aging-12-102641-s001..pdf]

SUPPLEMENTARY METHODS

Immunohistochemistry analysis

After paraffin sections were dewaxed and rehydrated, the antigen retrieval of hippocampus sections was performed using 99% formic acid (5 min at RT), following by heating-incubation (120°C for 20 min) with 10 mM Tris/1mM EDTA/0.05% Tween 20; pH 9.0. Endogenous peroxidase was blocked using 3% H_2O_2/methanol for 45 min. In a dark-humid chamber, blocking of sections was performed using Dako antibody diluent background reducing components (Dako) for 20 min, and treated with the Avidin/Biotin blocking kit from Vector Lab according to the manufacturer's instructions and incubated overnight (4°C) with antibodies against human Aβ, pathological phospho-tau (Ser202 and Thr205), NPC1, and StARD1 (Supplementary Table 2). Sections were then incubated (1 hr) with the corresponding biotinylated secondary antibodies diluted 1:200, followed by ABC staining for 30 min. The immunoreactivities were visualized with diaminobenzidine (DAB enhanced liquid substrate system, Sigma). Sections were counterstained with hematoxylin (Dako) and mounted with a coverslip. The final stained hippocampal sections were then scanned in a 43X resolution using Panoramic DESK digital slice scanner with Carl Zeiss objectives (3DHISTECH) and images of entire hippocampus regions where capture using Panoramic Viewer software (3DHISTECH).

Immunofluorescence analysis

Secondary antibodies (Thermofisher) were diluted 1:200 in 1% Goat Serum containing 0.05% Triton X-100 in PBS and incubated (1 hr at RT) with the mix of anti-rabbit Alexa fluor-532 (for StARD1 or NPC1), anti-mouse Alexa fluor-635 (for $A\beta_{42}$), and anti-rat Alexa fluor-488 (for GFAP). Finally, sections were incubated (10 min) with 20 mM Hoechst 33342 dye (Thermofisher), washed and incubated (5 min) with 0.1 % sudan black dye (Sigma) in 70% ethanol to block autofluorescence, followed by mounting using fluorescent mounting medium (Dako).

Human hippocampal cryopreserved 14 μm thin sections were fixed in 4% paraformaldehyde for 20 min. In a dark-humid chamber, the sections were permeabilized with 0.2% Triton X-100 in blocking buffer (5% goat serum + 1% BSA in PBS) for 2 hr. Then, slices were incubated (3 hr at 4°C) with the probe GST-PFO (20μg/ml) in 1% Goat Serum containing 0.05% Triton X-100 in PBS, followed by incubation overnight (4°C) with primaries antibodies (Supplementary Table 4). Secondary antibodies were diluted 1:200 in 1% Goat Serum containing 0.05% Triton X-100 in PBS and incubated for 90 min at RT with the mix of anti-rabbit Alexa fluor-647 (for Tom20 or Lamp1), anti-mouse Alexa fluor-532 (for GST) and anti-rat Alexa fluor-488 (for GFAP). After washing, slices were incubated 5 min in Sudan black 0.1% in 70% ethanol to minimize autofluorescence and mounted with prolong antifade mountant with Dapi (Dako).

Images for all samples were taken with a Leica TCS SP5 laser scanning confocal system with a 63X oil immersion objective APO CS numerical aperture 1.4 equipped with a DMI6000 inverted microscope.

Image analysis

Briefly, after background subtraction and filtering processes (median, radius 2.0), segmentation of immunoreactivities was performed by the combination of H-DAB vector matrix of *Color Deconvolution* plug-in [14] – which separate channels corresponding to 3 determined colors of hematoxylin-DAB stain – with the local standard threshold of the "*Colour-2*" (brown) resulted images. Immunoreactivities were then subjected to particle analysis for acquiring the percentage of immunolabeled area relative with the total area of the image.

Confocal images of immunoreactivities for GFAP, NPC1 and StARD1 from hippocampal paraffin sections were analysed by Image J Software. In 10 different images of each hippocampal region (CA1, CA2 or CA3), percentage of total NPC1 or StARD1 mass containing astrocytes (GFAP+) were analysed using the *Colocalization nBits n-images* plug-in (Confocal Microscopy Unit, Medicine Faculty, University of Barcelona), which highlights the colocalized points of two 32-bits images (Figure 4A). Two points are considered as colocalized if their respective intensities are strictly higher than the threshold of their channels and if their ratio of intensity is strictly higher than the ratio setting value, which have been defined at 50%. Colocalization index is calculated as the ratio of colocalized points between total threshold-passed intensity and relativized with control group.

On the other hand, similar to as been described above to paraffin sections, detection of cholesterol within lysosomes or mitochondria into astrocytes was performed in cryopreserved hippocampal sections and analysed by confocal imaging using Image J Software. Immunofluorescence images were submitted to background subtraction and filtering processes with Median filter set at 2.0 of radius. Then, 0.4 μm spaced

images were stacked at maximum intensity and segmented by threshold to select the area of astrocytes (GFAP+). All marks outside GFAP+ area were clean in the corresponding images for GST+ and Tom20+/Lamp1+. Resulted images were then analysed using the *Colocalization nBits n-images* plug-in (Confocal Microscopy Unit, Medicine Faculty, University of Barcelona) as is described above.

BRISQ checklist

Based on Moore et al, Cancer Cytopathol 2011; 119(2):92–101. https://doi.org/10.1002/cncy.20147.

Intracellular cholesterol accumulation associated with astrogliosis in postmortem brains from patients with AD and DS collected from from Biobank of Hospital Clinic (IDIBAPS) with the approval of the Clinical Research Ethics Committee of the Hospital Clinic of Barcelona (HCB/2015/0595).

**Biospecimen type**	**Brain sections**
**Anatomical site**	Cortex and Hippocampus
**Disease status of patients**	Alzheimer with or without Down Syndrome, Controls without neurological disease
**Clinical characteristics of patients**	Patients with Dementia
**Vital State of patients**	Postmortem
**Clinical diagnosis of patients**	Alzheimer dementia
**Pathology diagnosis**	CERAD B-C and Braak V-VI; detailed in Methods and in Table 1
**Collection mechanism**	Postmortem dissection
**Type of stabilization**	On ice
**Type of long-term preservation**	Formalin fixation, freezing
**Constitution of preservative**	10% neutral-buffered formalin
**Storage temperature**	−80 ºC, 20 to 25 ºC
**Storage duration**	5-10 years
**Shipping temperature**	−170 ºC to −190 ºC, 20 to 25 ºC
**Composition assessment and selection**	Brain sections used for anatomopathological diagnosis of Alzheimer
