# Supplementary material for: STARD1 and NPC1 expression as pathological markers associated with astrogliosis in post-mortem brains from patients with Alzheimer's disease and Down syndrome
Source: Aging (Albany NY). 2020 Jan 5;12(1):571–92. doi: 10.18632/aging.102641 (PMC6977657; doi:10.18632/aging.102641)
Supplement: Supplementary Figures [file aging-12-102641-s003..pdf]

SUPPLEMENTARY FIGURES

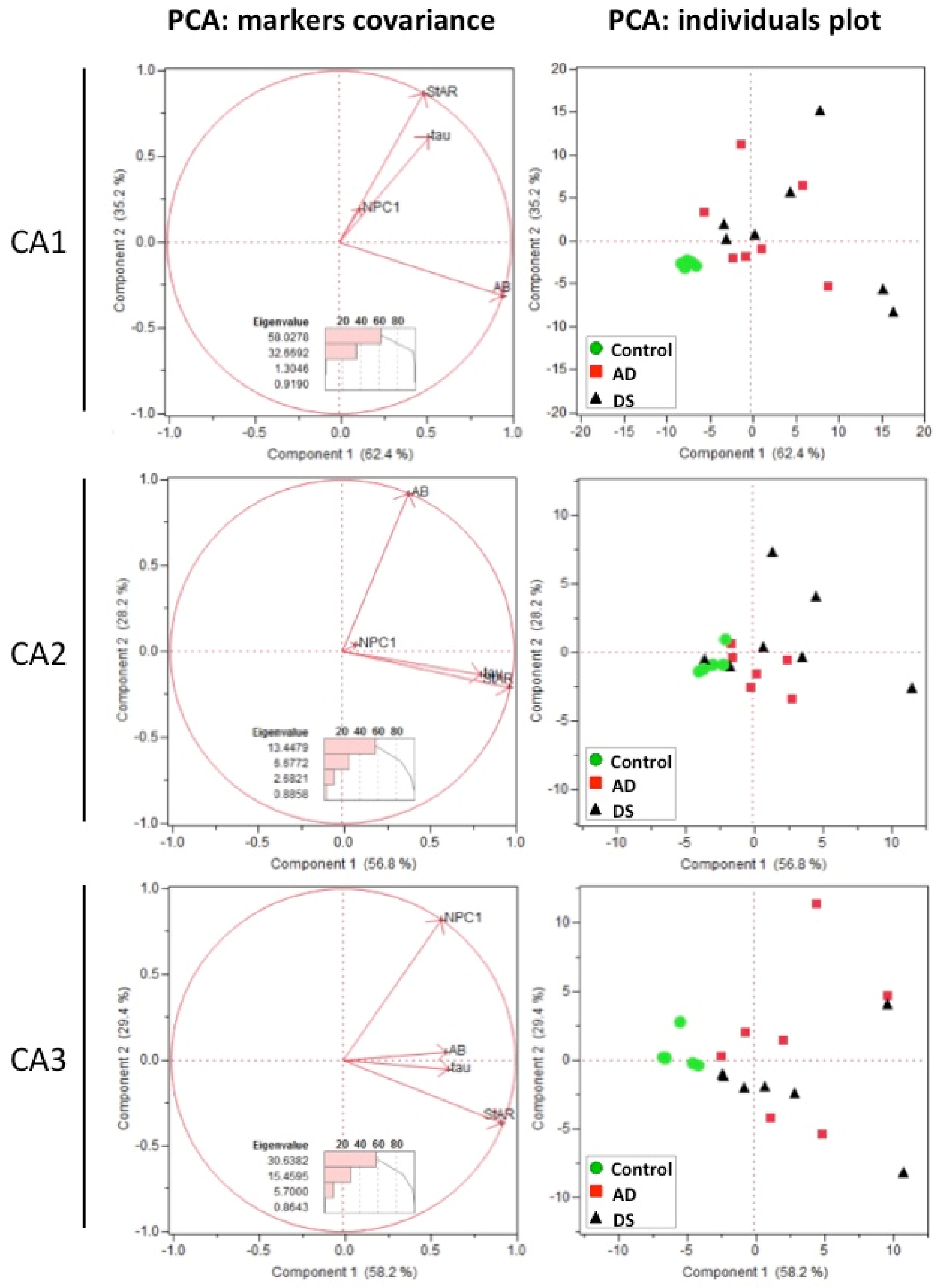

Supplementary Figure 1. Principal Component Analysis (PCA) of Aβ_{42}, p-tau, NPC1, and StARDl markers. For CA1, CA2, and CA3 hippocampus regions is show (left panel) the covariance between %area immunodetected by IHC of the corresponding marker, and the first principal plane (Component 1 + Component 2) represented on the first vectorial plan of the PCA. Histogram of Eigenvalues explain the variation. (Right panel) Distribution of AD, DS, and control samples onto the two first axes of the PCA.

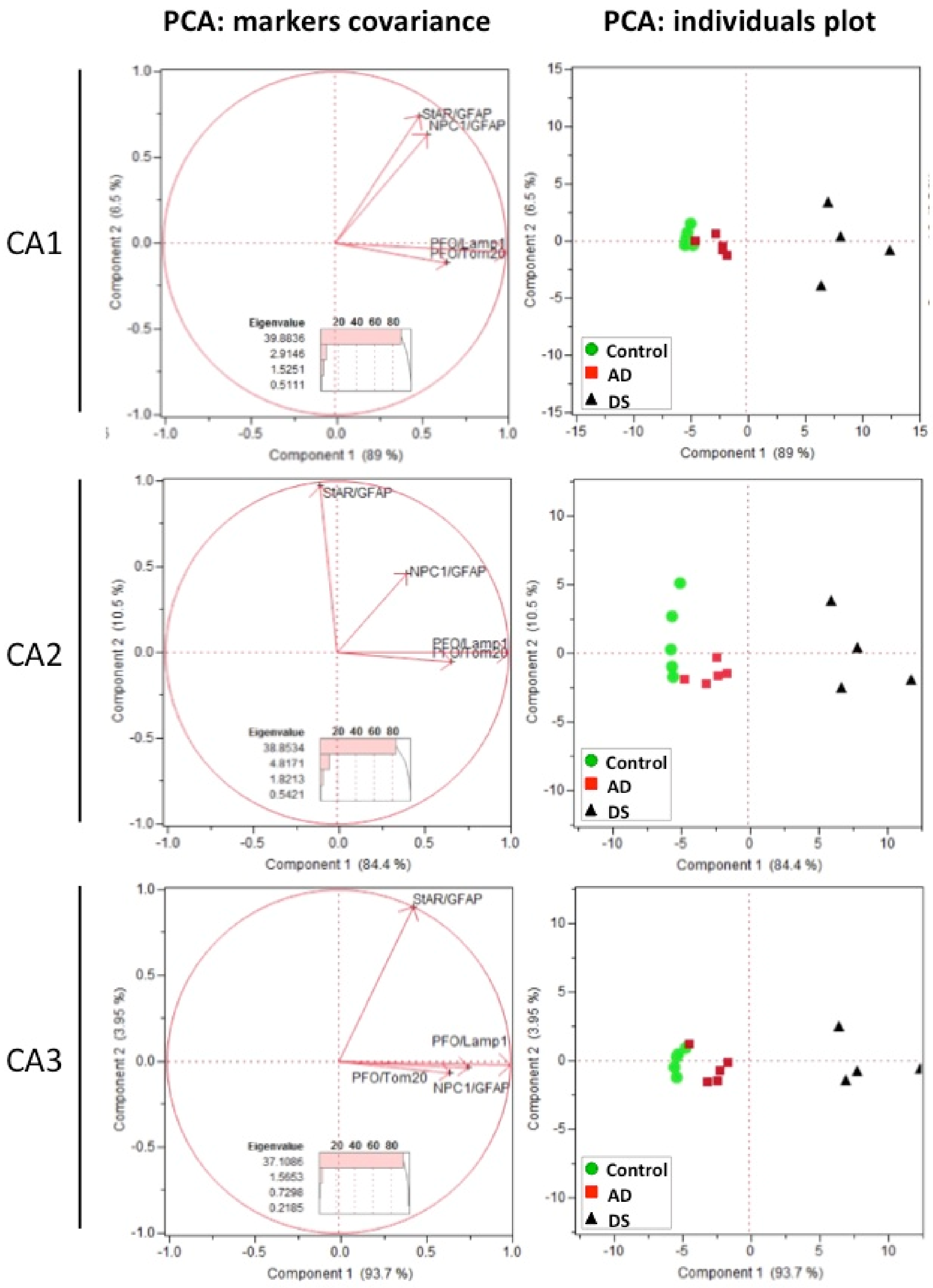

Supplementary Figure 2. Principal Component Analysis (PCA) of lysosomal and mitochondrial cholesterol contain and carriers within hippocampal astrocytes. (Left panel) First vectorial plan of the PCA that describe the covariance between descriptors (content of both glial cholesterol carriers and lysosomal/mitochondrial cholesterol content) and the first principal plane (Component 1 + Component 2). Histogram of Eigenvalues explain the variation. (Right panel) Distribution of AD, DS, and controlsamples onto the two first axes of the PCA.
