# Supplementary material for: STARD1 and NPC1 expression as pathological markers associated with astrogliosis in post-mortem brains from patients with Alzheimer's disease and Down syndrome
Source: Aging (Albany NY). 2020 Jan 5;12(1):571–92. doi: 10.18632/aging.102641 (PMC6977657; doi:10.18632/aging.102641)
Supplement: Supplementary Tables [file aging-12-102641-s002..pdf]

SUPPLEMENTARY TABLES

Supplementary Table 1. Overview of modulation of key proteins involved in intracellular cholesterol trafficking in the brain (1).

	Cortical levels (n-fold) *mean±SEM*		Hippocampal levels (n-fold) *mean±SEM*					
			CA1		CA2		CA3	
**Marker**	**mRNA**	**Protein[a]**	**Protein[b]**	**GFAP+ colocalization**	**Protein[b]**	**GFAP+ colocalization**	**Protein[b]**	**GFAP+ colocalization**
**Control**								
Aβ42	n.a.	n.a.	1.00 ± 0.262	n.a.	1.00 ±0.471	n.a.	1.00 ±0,323	n.a
p-tau	n.a.	n.a.	1.00 ±0.342	n.a.	1.00 ±0.368	n.a.	1.00 + 0.288	n.a.
NPC1	1.00 ±0.019	1.00 ± 0.017	1.00 ±0.173	1.00 ±0.196	1.00 ±0.357	1.00 ±0.120	1.00 ± 0.331	1.00 ±0.192
StARDI	1.00 ±0.015	1.00 ± 0.044	1.00 ±0.321	1.00 ±0.271	1.00 ±0.458	1.00 ±0.331	1.00 ±0.396	1.00 ±0.178
StARD3/ MLN64	1.00 ±0.030	1.00 ± 0.237	n.a.	n.a.	n.a.	n.a.	n.a.	n.a.
StARD4	1.00 ±0.023	n.a.	n.a.	n.a.	n.a.	n.a.	n.a.	n.a.
INSIG	n.a.	1.00 ± 0.053	n.a.	n.a.	n.a.	n.a.	n.a.	n.a.
SREBP2	n.a.	1.00 ± 0.028	n.a.	n.a.	n.a.	n.a.	n.a.	n.a.
**Alzheimer disease**								
Aβ42	n.a.	n.a.	7.04 ± 2.106	n.a.	1.97 ±0.727	n.a.	1.91 ±0.556	n.a.
p-tau	n.a.	n.a,	2.42 ±0.736	n.a.	1.63 ±0.554	n.a.	1.67 ±0.621	n.a.
NPC1	1.02 ±0.039	1.12 ±0.025 p_1=0.0043	8.07 ± 1.834 p_1=0.0006	0.92 ±0.194	3.77 ±0.800 p_1=0.0111	1.25 ±0.238	8.06 ± 1.539 p_1=0.0006	1.08 ± 0.087 p_2=0.0023
StARDI	1.00 ±0.014	1.25 ±0.056 P_1=0.0087	2.31 ±0.419 p_1=0.0379	2.60 ±0.563 P_1=0.0111	2.00 ±0.918	0.67 ± 0.232	5.92 ± 2.171 P_1=0.0047; p_2=0.0130	1.19 ±0.249
StARD3/ML N64	0.99 ± 0.062	0.64 ± 0.150	n.a.	n.a.	n.a.	n.a.	n.a.	n.a.
StARD4	1.00 ±0.020	n.a,	n.a.	n.a.	n.a.	n.a.	n.a.	n.a.
INSIG	n.a.	1.05 ± 0.160	n.a.	n.a.	n.a.	n.a.	n.a.	n.a.
SREBP2	n.a.	1.12 ±0.161	n.a.	n.a.	n.a.	n.a.	n.a.	n.a.
**Down syndrome**								
A(342	n.a.	n.a.	11.30 ±3.423 Pi=0.0012	n.a.	4.27 ±1.275 Pi=0.0177	n.a.	3.99 ± 1.051 Pi=0.0221	n.a.
p-tau	n.a.	n.a.	2.59 ±0.783	n.a.	2.29 ±0.784	n.a.	2.88 ± 1.135	n.a.
IMPCl	1.08 ±0.028	1.04 ±0.036	9.86 ±2.413 p_1=0.0006	1.62 ±0.420	5.41 ±1.740 P_1=0.0111	2.05 ± 0.577	8.79 ±1.822 P_1=0.0006	1.08 ± 0.087 p_1=0.0023
StARDI	1.08 ±0.013 P_1=0,0087	1.35 ±0.044 P_1=0.0043; p_2=0.0159	0.97 ±0.182 p_2=0.0111	2.81 ± 0.755	0.56 ±0.212	0.80 ± 0.246	2.62 ±1.290 p_2=0.0130	2.01 ±0.311 P_1=0.0111

StARD3/ MLN64	1.13 ±0.053	1.47 ±0.277	n.a.	n.a.	n.a.	n.a.	n.a.	n.a.
StARD4	1.03 ± 0.008	n.a.	n.a.	n.a.	n.a.	n.a.	n.a.	n.a.
INSIG	n.a.	1.14 ±0.043	n.a.	n.a.	n.a.	n.a.	n.a.	
SREBP2	n.a.	1.14 ±0.061	n.a.	n.a.	n.a.	n.a.	n.a.	n.a.

NOTE: P values are calculated by differences against control (pi) or differences between AD and DS (pz).

Supplementary Table 2. Overview of modulation of key proteins involved in intracellular cholesterol trafficking in the brain (1).

	Astrocyte (GFAP+) cholesterol (PFO+) colocalization (n-fold)		
Organelle marker	***Control***	***AD***	***DS***
Tom20 (mitochondria)	*1.00 ±0.257*	*2.55 ± 0.297; p_1=0.0079*	*2.77 ± 0.203; p_1=0.0159*
Lampl (lysosome)	*1.00 ±0.136*	*3.43 ± 0.502; p_1=0.0079*	*14.41 ± 1.322; $p_{1,2}$=0.0079*

Supplementary Table 3. Oligonucleotides used for gene expression analysis by RT-qPCR.

**Gene**	**Sequence**
StARD1	5′-GAGGAGGCCATGCAGAA<br>5′-GAACACCTTGCCCACATC
StARD3	5′-AGTGAGGAGCCCAGGGAG<br>5′-CCGTGGCTGACATGGAG
StARD4	5′-CGTTTTCTTAGCAACTCGCC<br>5′-CTTCCACGTCCTTGCTTCAC
NPC1	5′-CATCCTTTGGCAATGGTTTT<br>5′-CTGCTGCTACTGTGTCCAGC
β-Actin	5′-TTGCCGACAGGATGCAGAA<br>5′-GCCGATCCACACGGAGTACT

Supplementary Table 4. Primary antibodies used in this study.

Antibody	Source and type	Company	WB dilution	IHC/IF dilution
StARD1	Rabbit monoclonal	Abcam (ab133657)	1:1000	1:200
StARD3 (MLN64)	Rabbit polyclonal	Sant Cruz (sc-292868)	1:1000	
SREBP2	Rabbit polyclonal	Abcam (ab28482)	1:500	
INSIG-1	Rabbit polyclonal	Abcam (ab70784)	1:1000	
NPC-1	Rabbit polyclonal	Abcam (ab36983)	1:500	1:200
Aβ human (6F/3D)	Mouse monoclonal	Dako (M0872)		1:2000
Lamp1	Rabbit polyclonal	Sant Cruz (sc-5570)		1:200
Tom20	Rabbit polyclonal	Sant Cruz (sc-11415)		1:200
GFAP	Rat monoclonal	Calbiochem (345860)		1:200
Glutathione-S-Transferase (GST)	Mouse monoclonal	Sant Cruz (sc-374171)		1:200
PHF-tau (AT8)	Mouse monoclonal	Thermofisher (MN1020)		1:200
β-Actin-HRP	Mouse monoclonal	Sigma (A3854)	1:20000	
NeuN	Mouse monoclonal	Millipore (MAB377)		1:200
IBA1	Mouse monoclonal	Santa Cruz (sc-32725)		1:200
